# Responses to startling acoustic stimuli indicate that movement-related activation is constant prior to action: a replication with an alternate interpretation

**DOI:** 10.14814/phy2.12300

**Published:** 2015-02-06

**Authors:** Dana Maslovat, Ian M Franks, Alexandra Leguerrier, Anthony N Carlsen

**Affiliations:** 1 School of Kinesiology, University of British ColumbiaVancouver, British Columbia, Canada; 2 School of Human Kinetics, University of OttawaOttawa, Ontario, Canada; 3 Department of Kinesiology, Langara CollegeVancouver, British Columbia, Canada

**Keywords:** Movement-related activation, replication, response preparation, startle

## Abstract

A recent study by Marinovic et al. (J. Neurophysiol., 2013, 109: 996–1008) used a loud acoustic stimulus to probe motor preparation in a simple reaction time (RT) task. Based on decreasing RT latency and increases in motor output measures as the probe stimulus approached the “go” stimulus, the authors concluded that response-related activation increased abruptly 65 ms prior to the imperative stimulus, a result in contrast to previous literature. However, this study did not measure reflexive startle activity in the sternocleidomastoid (SCM) muscle, which has been used to delineate between response triggering by a loud acoustic stimuli and effects of stimulus intensity and/or intersensory facilitation. Due to this methodological limitation, it was unclear if the data accurately represented movement-related activation changes. In order to provide a measure as to whether response triggering occurred on each trial, the current experiment replicated the study by Marinovic et al., with the collection of muscle activation in the SCM. While the replication analyses involving all trials confirmed similar results to those reported by Marinovic et al., when data were limited to those in which startle-related SCM activation occurred, the results indicated that movement-related activation is constant in the 65 ms prior to action initiation. The difference between analyses suggests that when SCM activation is not considered, results may be confounded by trials in which the probe stimulus does not trigger the prepared response. Furthermore, these results provide additional confirmation that reflexive startle activation in the SCM is a robust indicator of response triggering by a loud acoustic stimulus.

## Introduction

In recent years, presenting a loud acoustic stimulus capable of eliciting a reflexive startle response during a reaction time (RT) task has been increasingly employed to examine the processes associated with movement preparation (for recent reviews see Valls-Solé et al. 2008; Carlsen et al. 2011b, 2012). Specifically, on trials where a startling acoustic stimulus (SAS) is presented, premotor RT (the time between the go-signal and the onset of muscle activation) is shortened to such a degree (i.e., <70 ms) that it is unlikely that the response was initiated via normal cortical stimulus–response processes. Rather, it has been hypothesized that the SAS acts as an involuntary trigger for the prepared movement (Valls-Solé et al. 1999; Carlsen et al. 2004b). If the processes of response preparation and initiation are considered within the context of a neural accumulator framework (e.g., Hanes and Schall 1996) in which neural activation increases to some subthreshold level prior to the go-signal (i.e., preparation), and then increases above threshold following the go-signal (i.e., initiation), then the response latency following the SAS allows for conclusions to be drawn regarding the preparatory state of the motor system at any given time (e.g., Maslovat et al. 2011, 2014a,b; Carlsen et al. 2012).

A recent study by Marinovic et al. (2013) used a loud acoustic stimulus to examine motor preparation prior to (Experiment 1) and following (Experiment 2) a visual imperative stimulus (IS) that was presented 1 s following a warning cue. Results showed that when the loud acoustic stimulus was presented 65 ms prior to the go-signal, premotor RT was longer than control RT values, with RT latency decreasing and motor output increasing as the stimulus was presented closer to the IS. Based on these data, the authors concluded that response-related activation increases abruptly in the final 65 ms prior to the go-signal. However, these results contrast with previous research showing that prepared movements can be triggered at a consistently short latency well in advance of the IS (i.e., 150–500 ms; MacKinnon et al. 2007; Carlsen and Mackinnon 2010; Alibiglou and MacKinnon 2012). Two explanations were proposed to explain this discrepancy: either participants were able to more accurately estimate the timing of the go-signal as compared to previous experiments and thus waited longer before preparing the response, or the intensity of the loud acoustic stimulus (114 dB) was insufficient to elicit the triggering of the prepared response at the earliest presentation times.

The determination as to whether the loud acoustic stimulus has triggered the response is often challenging as the observed RT decrease may be attributable to intersensory facilitation (Nickerson 1973), or simply to the well-documented response speeding effects of a more intense stimulus on the normal voluntary response processes (Woodworth 1938). Alternatively, the short latency response may be attributed to involuntary triggering via a separate mechanism that involves structures common to both the startle reflex and the prepared movement (Valls-Solé et al. 1999). One way to make this distinction is to examine the presence or absence of startle-related electromyographic (EMG) activity in the sternocleidomastoid (SCM) muscle on trials where a SAS is presented, as this indicates that the startle reflex circuitry was activated. Previous work has shown that when SCM activity is observed within 120 ms of the SAS, the latency to onset of the prepared response is consistently very short (∼80 ms) irrespective of the intensity of the stimulus (Carlsen et al. 2007). On these trials, activation of startle apparatus is associated with significantly decreased RTs, and thus is argued to be indicative of response triggering by the SAS. Conversely, on SAS trials where SCM activity is absent, RT is considerably longer (even at the highest intensities, e.g., 124 dB), more variable, and scales with stimulus intensity. These data suggest that in SAS trials lacking SCM activity, not all of these trials involve response triggering via subcortical mechanisms and may be confounded by stimulus intensity or intersensory facilitation effects on the normal cortical stimulus–response processes (Carlsen et al. 2007; see also Tresch et al. 2014). Therefore, the presence of short latency SCM activation on startle trials is a robust indicator of activity in startle-reflex-related structures that is sufficiently large to trigger the prepared response (see Carlsen et al. 2011b for more detail). In the study by Marinovic et al. (2013), SCM was not used as an indicator of startle; instead, activation in the orbicularis oculi (OOc) was used to determine if reflexive startle activity occurred. However, the OOc has shown to be a less reliable indicator of response triggering (Carlsen et al. 2007) as the eye blink response may, in some cases, reflect activity in a different circuit to that of the startle response (Brown et al. 1991; Kumru and Valls-Solé 2006; Carlsen et al. 2007).

The lack of collection of SCM data in the study by Marinovic et al. (2013) makes it difficult to determine whether the reported results are due (as suggested) to a late rise in movement-related activation or a lack of triggering by the acoustic probe stimulus. Confirmation that the observed response latency is due to consistent triggering of the prepared movement by the SAS is particularly important in studies (such as Marinovic et al. 2013) where the SAS is presented *prior* to the IS with instructions given to only respond to the visual “go” signal (not the SAS). By not separating SAS trials depending on whether or not a startle response in the SCM was observed, it remains unknown if the response was being involuntarily triggered by the SAS via a subcortical mechanism, or voluntarily initiated in response to the IS (as instructed). As the IS and SAS are separated in time, this information is critical in order to infer preparatory activation level based on RT latency. One indication that participants in the Marinovic et al. study may have been responding to the IS, at least on some trials, is that loud stimulus-referenced RT values when presented at −65 ms with respect to the go-signal were longer and more variable than those in control trials; a particularly unusual result for startle trials (e.g., Valls-Solé et al. 1995, 1999; Carlsen et al. 2004a,b). This would not be expected if the SAS was triggering the prepared response but would be expected if some trials involved participants responding to the IS, while other trials involved response triggering by the SAS. This explanation is also worthy of exploration as the 114 dB auditory stimulus used by Marinovic et al. has been shown to elicit SCM activation in approximately one half of trials (Carlsen et al. 2007, 2009), and is considerably lower than that used in many previous studies that have used a SAS to investigate response preparation (124–130 dB) (e.g., Valls-Solé et al. 1999; Carlsen et al. 2004b; Marinovic et al. 2014; Tresch et al. 2014).

The purpose of this study was to replicate *Experiment 1* of Marinovic et al. (2013), but with the added collection and analysis of EMG activity in the left SCM muscle. Similar to previous work (Carlsen et al. 2007, 2011b; e.g., Honeycutt and Perreault 2012; Honeycutt et al. 2013; Marinovic et al. 2014; Tresch et al. 2014), classification of SAS trials was performed on the basis of presence or absence of SCM activation (SCM+ or SCM−), to allow for delineation between response triggering by the loud acoustic stimulus and either stimulus intensity effects associated with a loud acoustic probe or intersensory facilitation effects associated with the auditory probe coupled with the visual IS. A comparison of the results of the analyses of all trials to those separated by SCM activation allows for a more critical examination of the conclusions offered by Marinovic et al. to determine if the observed RT differences were indeed due to movement-related activation differences or a lack of sufficient stimulus intensity to consistently elicit a triggered response.

## Materials and Methods

### Participants

Data were collected from 15 right-handed volunteers with normal or corrected-to-normal vision. Data from one participant were excluded due to a high proportion of false-start (i.e., anticipation) errors (16% of trials), while that of a second participant was excluded due to very slow responses (control RT was greater than three standard deviations above the between-participant mean). Thus, data are presented from 13 participants (9 M, 4 F; 24.3 ±6.0 years). All participants provided informed consent prior to the start of the experiment and were naïve to the hypotheses under investigation. The study was conducted in accordance with ethical guidelines established by the Research Ethics Board at the University of Ottawa and conformed to the latest revision of the Declaration of Helsinki.

### Apparatus and task

The apparatus and task parameters used here replicate as closely as possible those implemented by Marinovic et al. (2013). Participants were required to make a brief isometric flexion of the right wrist against a force transducer (Tekscan FlexiForce A201) in response to the appearance of a visual IS presented on the computer screen as a bright green circle (3 cm diameter). The IS was preceded by a warning stimulus consisting of a red circle (3 cm diameter), with a constant foreperiod of 1 sec. On 10% of trials, no IS was presented (catch trials). On another 10% of the trials, a loud acoustic stimulus was delivered binaurally via headphones (Sennheiser Model HD280 Pro) at time intervals before (−65, −40, and −15 ms), concurrent with (0 ms), and following (+15 ms) the IS. The loud acoustic stimulus consisted of a 50-ms duration broadband noise pulse (10 Hz–30 kHz), amplified to produce a peak intensity of 114 dB. Stimulus intensity was calibrated and verified using a precision sound level meter (Cirrus Research CR:162C), “A”-weighted peak response mode, measured 2 cm from the headphone speaker.

Participants were seated ∼0.8 m away from a 23” LCD computer monitor that was used to present the visual stimuli and provide feedback. On control trials, feedback consisted of RT presented on the screen for 3 sec. If RT was shorter than 110 ms, a message was displayed instructing the participant to wait until appearance of the IS. On catch trials, the message informed participants whether or not they were successful in refraining from responding. No feedback was displayed on trials in which the loud acoustic stimulus was delivered. Trial presentation and data collection were performed using customized LabVIEW® software with a temporal resolution of <1 ms.

Surface EMG data were collected using preamplified surface electrodes connected via shielded cabling to an external amplifier system (Delsys Bagnoli-8). Recording sites were prepared and cleansed in order to decrease electrical impedance, with electrodes attached parallel to the muscle fibers using double-sided adhesive strips. A grounding electrode was placed on the right radial styloid process with data collected from right flexor carpi radialis (FCR), left orbicularis oculi (OOc), and left SCM. EMG and force data were sampled at 4000 Hz via a National Instruments data acquisition device (PCIe-6321; National Instruments, Austin, TX) for a total of 3000 ms, starting 500 ms prior to the IS.

### Design and procedures

Participants were provided with an initial practice block of 40 trials, including 36 control trials and four catch trials. Practice was followed by four, 100 trial testing blocks with each block including 10 catch trials, 10 trials in which the auditory stimulus was presented twice at each of the five time intervals, and 80 control trials. This resulted in a total of 40 catch trials, 40 probe trials (eight presented at each of the time intervals), and 320 control trials. Throughout the experiment, catch trials and auditory probe trials were presented pseudorandomly such that there were never consecutive catch or auditory trials. Participants were instructed to respond only to the IS while avoiding false starts on catch trials (false starts occurred on 10% of trials). Instructions were given to produce a “comfortable, brief, and stable exertion of force” and no feedback about the amount of force was provided, unless it was insufficient to be detected.

### Data reduction

Data reduction was completed using customized LabVIEW® software. EMG data were full-wave rectified, dual passed filtered using a 25 Hz low-pass second-order elliptical filter, with a Teager-Kaiser energy operator (TKEO) (Li and Aruin 2005) applied to improve the detection of onset of muscle activity. EMG burst onsets for both agonist and startle indictors were defined as the point at which TKEO transformed EMG activity reached a value of seven standard deviations above baseline levels (mean activity from −500 to −400 ms) and remained above that level for more than 20 ms. EMG offsets were defined as the first point following onset in which activation dropped below 20% of peak EMG and remained below that level for more than 20 ms. Force onset and offset were determined in a similar fashion with a minimum change of 0.1 N required from baseline. All markers were visually confirmed and manually adjusted (if necessary) to compensate for any errors due to the strictness of the algorithms.

### Replication analysis

In order to replicate the analyses performed by Marinovic et al. (2013), the dependent variables of premotor RT (time between IS or auditory stimulus and EMG onset in FCR muscle), peak force (maximal force between force onset and offset, expressed a proportion of the mean of control trial values), time to peak force (time between force onset and peak force), force duration (time between force onset and offset), peak EMG (maximal FCR EMG, expressed a proportion of the mean of control trial values), and EMG duration (time between FCR EMG onset and offset) were calculated. Each dependent measure was initially subjected to a repeated measures one-way analysis of variance (ANOVA), with six levels of condition (control, −65, −40, −15, 0, +15). Differences between control trials and acoustic stimulus trials were assessed using post-hoc Bonferroni corrected *t*-tests. One-way repeated measures ANOVAs were also performed without control trials to examine any significant trends in acoustic stimulus trials with respect to lead time, using polynomial contrasts adjusted for unequal time intervals.

### Reanalysis using startle indicators

In addition to the replication analyses, the proportion of acoustic stimulus trials in which EMG activity was detected in OOc and SCM (within 120 ms of the auditory stimulus; Carlsen et al. 2011b) as an indication of a startle response, was examined via a 2 Indicator (SCM, OOc) × 5 Condition (−65, −40, −15, 0, +15 ms) repeated measures ANOVA. Furthermore, a direct comparison of all dependent measures was performed comparing acoustic stimulus trials where a burst of EMG was present in the startle indicator, with those where no EMG burst was evident (i.e., SCM+ vs. SCM−; OOc+ vs. OOc−), to determine the effect of the presence or absence of each startle indicator. However, due to the small number of SAS trials that resulted in SCM activation (i.e., SCM+) using the 114 dB stimulus, as well as the low number of SAS trials that lacked OOc activation (i.e., OOc−) (i.e., less than 1/3 of trials in each case; see “Startle indicators” section in results below), many participants did not have a mean value for each condition, thus it was not possible to perform a repeated measures ANOVA. To overcome this limitation, a univariate ANOVA was performed on each dependent measure using each trial as an observation, rather than using subject mean values. This resulted in what can be considered a between-group analysis, even though all data came from a single set of subjects, with fixed factors including two levels of Startle Indicator (present, absent) and five levels of Condition (−65, −40, −15, 0, +15 ms). As each trial represented an observation, outliers were removed by excluding those trials in which premotor RT values exceeded two standard deviations from the overall between-participant mean for each condition (13/147 for SCM+, 17/358 for SCM−, 16/347 OOc+, 9/158 OOc−).

Previous investigation of the startle reflex itself has shown differences in the expression of the startle response depending on the indicator. For example, OOc does not readily habituate with repeated SAS stimulation, whereas SCM quickly habituates (Brown et al. 1991; Valls-Sole et al. 1997; Kumru and Valls-Solé 2006). This has led Brown et al. (1991) to suggest a two-component model of the eyeblink response to loud acoustic stimuli: a non-startle-related auditory blink response (which does not habituate), and a separate component involving a startle-related blink reflex (which does show habituation). This distinction was supported by data from Carlsen et al. (2007) who found that OOc activity was substantially increased when SCM activation was also present, presumably due to the presence of the startle reflex blink response. To examine if OOc activation differences were present in the current experiment as a function of SCM presence, we performed an univariate ANOVA on OOc onset, duration, and peak activation for those trials in which OOc activation was observed, via a 2 SCM indicator (present, absent) × 5 Condition (−65, −40, −15, 0, +15 ms) univariate ANOVA. The alpha level for the entire experiment was set at 0.05.

## Results

### Replication analysis

#### EMG measures

**Figure 1:**
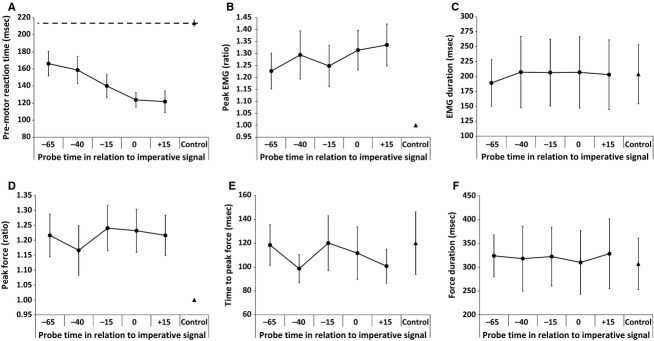
Mean data (SEM error bars) from the replication analyses, including EMG and force measures based on acoustic probe times. Probe trials are represented by circles, while the control data are represented by a triangle. Note the significant linear decrease in premotor reaction time (panel A) and linear increase in peak EMG (panel B) as the probe time approaches the imperative signal.

Analysis of premotor RT data for probe and control trials indicated an effect of condition, *F*(5, 60) = 12.66, *P *<* *0.001, 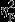 = 0.51, which post-hoc analyses confirmed that premotor RT was significantly longer on control trials as compared to when the acoustic stimulus was presented at −15, 0 and +15 ms. Analysis excluding the control trials also revealed a significant linear trend, *F*(1, 12) = 12.31, *P *=* *0.004, 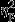 = 0.51, indicating that premotor RT decreased progressively as the time interval approached the IS (Fig.1A). Peak EMG analysis also indicated an effect of condition, *F*(5, 60) = 7.49, *P *<* *0.001, 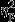 = 0.38, with post-hoc analyses showing a significant difference between peak EMG in control trials and probe trials at both 0 ms and +15 ms. Analysis excluding the control trials showed a significant linear trend, *F*(1, 12) = 5.01, *P *=* *0.045, 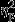 = 0.29, indicating that peak EMG progressively increased as the time interval approached the IS (Fig.1B). EMG duration (Fig.1C) was not affected by condition, *F*(5, 60) = 0.49, *P *=* *0.783, 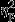 = 0.04, and did not show a significant linear trend with control trials excluded, *F*(1, 12) = 0.56, *P *=* *0.692, 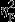 = 0.05.

#### Force measures

Analysis of peak force indicated an effect of condition, *F*(5, 60) = 6.09, *P *<* *0.001, 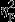 = 0.34; however, post-hoc analyses using the strictness of a Bonferroni correction found no differences. Visual inspection of the data (Fig.1D) shows a lower control value as compared with all probe conditions, which was confirmed using a less conservative Fisher's LSD post-hoc analysis. Analysis of peak force excluding the control trials did not result in a significant linear trend, *F*(1, 12) = 0.97, *P *=* *0.343, 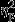 = 0.08. For time to peak force (Fig.1E), no effect of condition was found, *F*(5, 60) = 1.33, *P *=* *0.266, 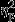 = 0.10, nor a significant linear trend, *F*(1, 12) = 0.86, *P *=* *0.372, 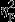 = 0.07. Similarly, force duration (Fig.1F) showed no effect of condition, *F*(5, 60) = 0.271, *P *=* *0.927, 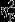 = 0.02, or a significant linear trend, *F*(1, 12) = 0.00, *P *=* *0.987, 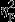 = 0.00.

#### Summary

Overall, data from this study closely replicated those reported in the study by Marinovic et al. (2013). When the data were analyzed without consideration to whether or not a startle indicator was present, premotor RT systematically decreased as the probe time approached the IS, with probe times exhibiting shorter values than control trials. Similarly, peak EMG progressively increased as the probe time approached the IS, with values larger than control trials. Also, consistent with the reported results by Marinovic et al., no differences were found in EMG duration or time to peak force.

### Reanalysis using startle indicators

#### Startle indicators

The analysis of the proportion of trials with an acoustic stimulus in which a burst of EMG activity occurred in either the SCM or OOc, showed a main effect for indicator, *F*(1, 12) = 7.71, *P *=* *0.017, 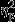 = 0.39, due to a significantly lower proportion of trials in which SCM activation occurred (28%) as compared to OOc (69%). No effect was found for condition, *F*(4, 48) = 0.66, *P *=* *0.625, 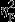 = 0.05, and there was no interaction effect, *F*(4, 48) = 1.21, *P *=* *0.318, 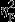 = 0.09.

#### Effect of SCM activation

**Figure 2:**
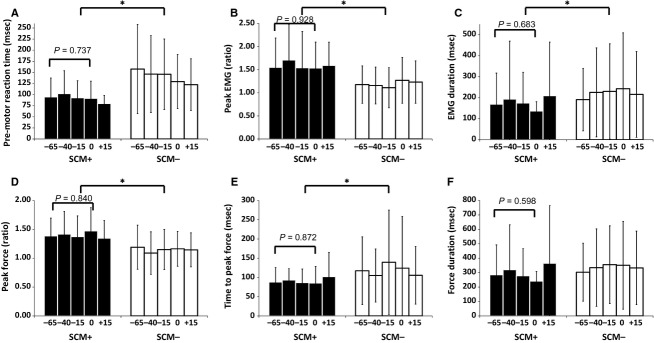
Mean data (SD error bars) for all dependent measures, based on acoustic probe lead time, separated by whether SCM activation was present (SCM+, black bars) or absent (SCM-, white bars). An asterisk (*) denotes a significant effect between SCM+ and SCM− trials. SCM+ trials were performed with significantly shorter reaction time (panel A), higher peak EMG (panel B) and force (panel D), and shorter time to peak force (panel E) and EMG duration (panel C). Note that lead time did not significantly affect any dependent measures, with *P* values provided for the post-hoc analysis of SCM+ trials from −65 ms to the imperative stimulus.

The comparison of dependent measures for each probe time, separated by presence or absence of activation in the SCM (SCM+/SCM−), is shown in Figure2. All dependent measures except for force duration [*F*(1, 465) = 2.07, *P *=* *0.150, 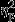 = 0.04], showed a main effect of SCM presence. On trials where SCM activation was observed, premotor RT was significantly shorter [*F*(1, 465) = 56.93, *P *<* *0.001, 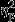 = 0.11], peak EMG activation [*F*(1, 465) = 51.28, *P *<* *0.001, 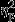 = 0.10], and peak force [*F*(1, 465) = 57.48, *P *<* *0.001, 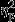 = 0.11] were significantly higher, while time to peak force [*F*(1, 465) = 8.62, *P *=* *0.003, 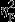 = 0.02] and EMG duration [*F*(1, 465) = 4.31, *P *=* *0.039, 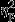 = 0.01] were shorter. There was no significant main effect of condition or interaction between SCM presence and condition for any of the dependent measures, indicating that probe lead time did not have an effect on the EMG or force measures for SCM+ or SCM− trials.

Although the lack of a significant effect of probe lead time on all dependent measures would suggest a similar level of preparatory activation prior to the IS, a nonsignificant finding does not ensure the independent measure had no effect on performance. Thus, a post-hoc analysis was performed to more closely determine if any differences in performance were apparent on the SCM+ trials, as these were hypothesized to most accurately represent trials in which response triggering occurred and thus would be indicative of preparatory-related activation levels. The post-hoc analysis involved a univariate ANOVA on the time points from −65 ms to the IS presentation (0 ms) for SCM+ trials only. This follow-up analysis provided additional confirmation that activation levels were constant prior to the IS as none of the dependent measures approached a significant effect of probe lead time and all effect sizes were minimal: premotor RT, *F*(3, 105) = 0.42, *P *=* *0.737, 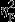 = 0.01; peak EMG, *F*(3, 105) = 0.15, *P *=* *0.928, 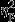 = 0.00; EMG duration, *F*(3, 105) = 0.50, *P *=* *0.683, 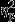 = 0.01; peak force, *F*(3, 105) = 0.28, *P *=* *0.840, 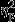 = 0.01; time to peak force, *F*(3, 105) = 0.24, *P *=* *0.872, 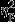 = 0.01; force duration, *F*(3, 105) = 0.63, *P *=* *0.598, 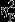 = 0.02.

The analysis examining the OOc burst characteristics when SCM activation was present or absent provided further evidence that the presence of SCM activation resulted in a quantitatively different response in not only the prepared muscles but also in other startle indicators. On SCM+ trials, OOc activation occurred a significantly earlier onset [*F*(1, 311) = 4.25, *P *=* *0.040, 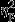 = 0.02] with greater peak activation [*F*(1, 311) = 59.49, *P *<* *0.001, 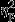 = 0.16], while duration was unchanged [*F*(1, 311) = 0.30, *P *=* *0.589, 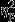 = 0.00].

#### Effect of OOc activation

**Figure 3:**
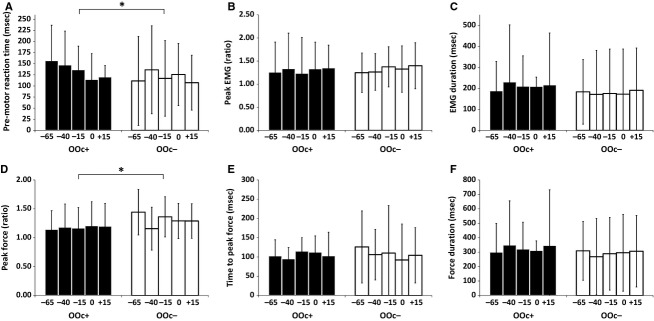
Mean data (SD error bars) for all dependent measures, based on acoustic probe lead time, separated by whether OOc activation was present (OOc+, black bars) or absent (OOc−, white bars). An asterisk (*) denotes a significant effect between OOc+ and OOc− trials. OOc+ trials were performed with significantly longer reaction time (panel A) and lower peak force (panel D). Note that lead time did not significantly affect any dependent measures.

The comparison of dependent measures for each probe time, separated by presence or absence of activation in the OOc (OOc+/OOc−), is shown in Figure3. In contrast to the presence of SCM activation, only two dependent measures showed a significant effect depending on whether OOc activation was present or absent; however, these effects were in the opposite direction to those expected by response triggering. That is, on trials where OOc activation was observed, premotor RT was significantly longer [*F*(1, 470) = 4.32, *P *=* *0.038, 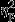 = 0.01], and peak force was significantly lower [*F*(1, 470) = 12.37, *P *<* *0.001, 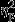 = 0.03] compared to trials where no OOc activity was observed. Similar to the SCM+/− analyses, no dependent measures showed a significant effect of condition or an interaction between OOc presence and condition, indicating that probe lead time did not affect any of the EMG or force measures for OOc+ or OOc− trials.

#### Summary

When consideration was given to whether or not a startle indicator was present a different pattern of results emerged. Although comparatively few loud acoustic stimulus trials resulted in SCM activation (28%), these trials differed on almost all dependent measures when compared to trials without SCM activation. This included significantly shorter premotor RT as well as higher peak force and peak EMG. Although a higher proportion of trials resulted in OOc activation (69%), these trials resulted in longer premotor RT and a lower peak force. Lastly, no dependent measures for either startle indicator were affected by probe lead, indicating that performance was similar during the time period from 65 ms prior to the IS.

## Discussion

The purpose of this experiment was to use a loud acoustic stimulus to examine movement-related preparatory activation in the time period immediately preceding the IS in a simple reaction time task. Previous research has indicated that presenting a 124 dB SAS in RT tasks results in a high rate (∼90%) of early response triggering if presented in a 500 ms window prior to the IS (e.g., Carlsen and Mackinnon 2010; Alibiglou and MacKinnon 2012), suggesting a high level of response-related activation is maintained for a relatively long period prior to response initiation, even when the time of the required response is somewhat predictable (Carlsen and Mackinnon 2010). These results are also in line with a model of activation in premotor areas proposed by Thickbroom et al. (2000; see also Carlsen and MacKinnon 2010; Carlsen et al. 2012). However, more recent work by Marinovic et al. (2013) has challenged this notion by showing that a loud acoustic stimulus presented in the final 65 ms prior to the IS led to a significant reduction in RT and increase in motor output as the probe approached the IS. This result was used as evidence for increasing activation occurring in the late stages of action anticipation, and was suggested to have occurred due to an ability to accurately predict the appearance of the IS.

The results of this study replicated and confirmed the data presented by Marinovic et al. (2013), showing that when data from all trials are considered, RT latency systematically decreases prior to the IS (Fig.1A)^1^ and peak EMG progressively increases prior to the IS (Fig.1B). However, this result changes drastically when trials are classified based on the presence or absence of reflexive startle activation in the SCM (Fig.2), which has been shown to be a robust indicator that can delineate between trials where voluntary RT is facilitated by stimulus intensity or intersensory facilitation, as compared to trials resulting in early and involuntary response triggering via subcortical circuits (Carlsen et al. 2007). On trials where SCM activation was present (SCM+), response latency was short and consistent at all probed time points, whereas the RT of trials without SCM activation (SCM−) were significantly longer and more variable (Fig.2A). Similarly, peak EMG was significantly larger on SCM+ trials and was not affected by probe time (Fig.2B). These results provide strong indication that movement-related activation is already at a greatly heightened state and is held at a consistently high level in the 65 ms prior to the IS, consistent with the neural activation model proposed by Carlsen et al. (2012). In addition to premotor RT and peak EMG, trials in which SCM activation was present showed significantly shorter EMG duration (Fig.2C), higher peak force (Fig.2D), and shorter time to peak force (Fig.2E). Given the contrast between results for SCM+ and SCM− trials, it is clear that the presence of SCM results in a different response that is more indicative of the response triggering effect of a SAS. Further evidence for SCM activation being an indicator of a different neural process is shown by the change in eyeblink response when an SCM burst was observed. During SCM+ trials, OOc activation occurred earlier with higher peak activation, denoting that the presence of SCM activation is related to changes in both the prepared muscles and other startle indicators. Note that these effects occurred when SCM activation was present, even though the probe stimulus was identical for all trials, indicating that these results are not due to stimulus intensity or intersensory facilitation, but rather to the presence or absence of startle-related activation in the SCM.

Although OOc activation is often used as an indicator of reflexive startle activity (Davis 1984; Blumenthal et al. 2005), results from this study clearly show that SCM activation is a more consistent indicator of a sufficiently strong startle response to lead to the early triggering of a prepared voluntary action via subcortical circuits. Marinovic et al. (2013) used the eyeblink response to determine if reflexive startle activity occurred; however, previous research has indicated that OOc is less reliable than SCM as a startle indicator with respect to the triggering of prepared actions (Carlsen et al. 2007). Indeed, in this study, delineation between OOc+ and OOc− trials did result in differences in premotor RT (Fig.3A) and peak force (Fig.3D); however, the presence of OOc activation resulted in longer RT with reduced peak force, opposite to the expected results of involuntary response triggering associated with a SAS. Based on the current data, including both the replication analyses (Fig.1) and those involving separation of trials by SCM (Fig.2) and OOc (Fig.3) activation, we argue that the conclusions from Marinovic et al. are likely confounded by a lack of consistent response triggering by the probe stimulus. Although discarded by the authors, this possibility was suggested as a potential explanation for their results that contrasted previous research involving a SAS delivered prior to IS.

The use of a loud acoustic stimulus with a visual IS poses particular challenges for data interpretation as a decreased RT latency can be confounded by stimulus intensity (Woodworth 1938) and intersensory facilitation (Nickerson 1973) effects. However, both effects are thought to be due to faster voluntary response initiation processes as stimulus intensity effects have been attributed to less time to reach a stimulus identification threshold (e.g., Grice 1968; Kohfeld 1971) and intersensory facilitation effects have been attributed to the additive interaction of visual and auditory response processes (e.g., Nickerson 1973; Miller 1982; Gielen et al. 1983). Conversely, the involuntary response triggering effect of startle is suggested to involve different neural circuits that are common with the startle reflexive response (e.g., Valls-Solé et al. 1999; Carlsen et al. 2004a), which has been supported by a number of studies in the startle literature. For example, Carlsen et al. (2007) manipulated stimulus intensity and showed that trials without a startle indicator exhibited typical stimulus intensity effects (from ∼120 ms at 83 dB to ∼100 ms at 123 dB), whereas SCM+ trials were performed at a significantly lower RT latency (∼80 ms) at all stimulus intensities. With regard to intersensory facilitation, while pairing an auditory tone with visual IS has been shown to speed RT (i.e., displacement RT shortened from 220 ms to 204 ms; Gielen et al. 1983), latencies are still well within voluntary initiation range. Furthermore, in contrast to the predictions of intersensory facilitation, pairing a SAS with a visual IS does not result in a further reduction in RT latency, providing additional support for a separate, faster neural pathway (Carlsen et al. 2011a). Although startle trials typically produce RT latencies that are below what would be considered possible through voluntary initiation channels, recent evidence has been provided that startle trials can exhibit delayed RTs due to lowered levels of preparation (Maslovat et al. 2013, 2014b). Thus, response latency alone cannot be used to differentiate between response triggering, stimulus intensity, and intersensory facilitation effects. Instead, an independent validation of a reflexive startle response is required, which the current and previous data suggest that can be provided by startle-related activation in the SCM.

**Figure 4:**
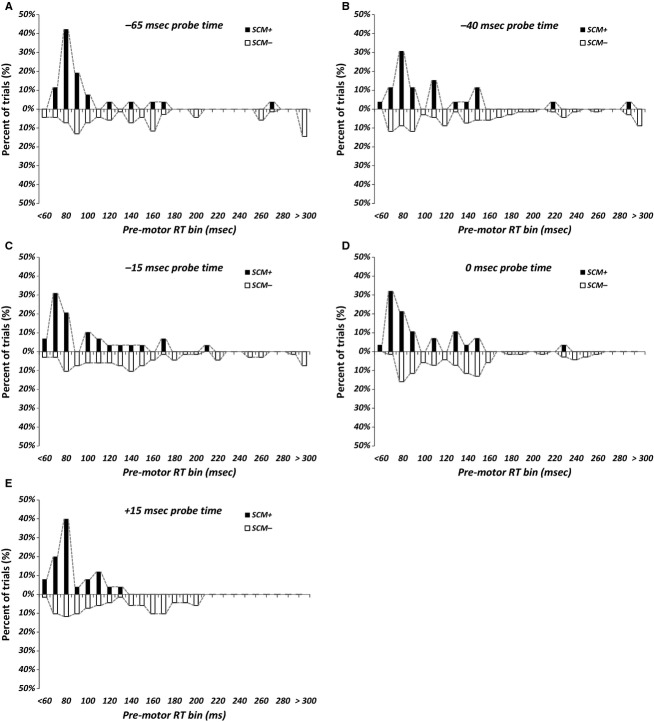
Premotor reaction time (RT) distribution in 10 ms bins, separated by those trials with SCM activation (SCM+, black bars) and without SCM activation (SCM−, white bars), for each probe time (panels A through E). Note that for SCM+ trials, the majority of RT distribution lies in the 60–100 ms range (indicative of response triggering), while SCM− trials show a greater distribution in the 100–300 ms range (indicative of voluntary initiation processes).

While the current data suggest that the presence of SCM activity confirms sufficient subcortical reflexive activation to result in response triggering, few trials (28%) reached this criterion using the 114 dB stimulus. In order to better understand the relative effects contributing to RT differences between SCM+ and SCM− trials, data were separated into 10 ms RT distribution bins and plotted by condition (Fig.4). At all probe times, the distribution of SCM+ trials is predominantly made up of trials with RTs ranging from 60 to 100 ms (black bars). Conversely, while there are some SCM− trials with RTs in the 60–100 ms range, there is a considerable scattering of trials with RTs in the 100–300 ms range (white bars). Thus, it appears that in SCM− trials, while there may be some RTs that occur at a response latency that would be indicative of involuntary response triggering, many other RTs show a latency that is more representative of voluntary initiation processes. In comparison, when SCM activity is present (SCM+) there are very few trials where RTs would be considered to be in the normal voluntary initiation range. These results indicate that when SCM activation is present, it is more likely that response triggering has occurred but when SCM activation is absent, the response *might* have been triggered by the SAS, *or* it may reflect a voluntary response to the IS. This conclusion is further supported by examination of the SCM− distribution of RTs as the probe time approaches the IS. As the time separation between the probe and IS becomes less, the distribution of trials at long latencies (>200 ms) becomes smaller, as would be expected when the auditory probe is either simultaneous with (Fig.4D) or immediately following (Fig.4E) the visual IS.

Although previous work has suggested that reflexive SCM activation is directly related to response triggering (Carlsen et al. 2007), few studies have replicated these results and examined in detail how the presence or absence of various startle indicators is related to task performance. By comparing both SCM+ versus SCM− (Fig.2) and OOc+ versus OOc− (Fig.3) trials, the current data confirm that the presence of SCM activation results in shorter latency responses that are consistent with response triggering effects. While it is apparent from examination of the RT distributions (Fig.4) that this distinction is not absolute, it is equally apparent that SCM− trials include a greater number of long-latency responses that are not typically associated with response triggering. The explanation offered for the results of the SCM− trials is that other effects such as stimulus intensity and intersensory facilitation may be occurring on individual trials, which involved a different, slower, voluntary process. One option to reduce this confound is to increase the intensity of the auditory stimulus as the proportion of SCM+ trials scales with stimulus intensity (Carlsen et al. 2007). Although the current data still suggest that SCM− trials should be removed from any analysis, a > 120 dB startling stimulus should result in a high percentage of SCM+ trials (e.g., 90–100%; Carlsen and Mackinnon 2010) as compared to the 28% SCM+ trials when using a 114 dB stimulus, as in this study. Finally, the assertion that SCM+ trials are representative of different neural pathways is not limited to the use of a SAS. The examination of long-latency responses to an external perturbation has revealed that when participants prepare a response to the perturbation, reflexive activity in the SCM is consistently elicited, along with a corresponding decrease in response latency of the planned movement (Ravichandran et al. 2013). Conversely, when no response is planned, SCM activity is not observed, nor is early activation of the involved muscles. This result has been taken as supporting evidence that SCM activation is directly related to triggering a prepared response via startle-related neural circuits, rather than reflexive activation or stimulus intensity effects.

In summary, the data collected in this study are consistent with those reported by Marinovic et al. (2013), which were used to conclude that movement-related activation increase in anticipation of action. However, when data are limited to trials in which startle-related SCM activation occurs, the results indicate that movement-related activation is in fact constant 65 ms prior to action initiation. This is consistent with previous work examining preparatory levels before an IS, and suggests that the results reported by Marinovic et al. were likely confounded by trials in which the probe stimulus did not trigger the prepared response. These results also further confirm the relationship between reflexive startle-related activation in the SCM and the triggering of a prepared response by a loud acoustic stimulus.

## Conflict of Interest

The authors declare that the research was conducted in the absence of any commercial or financial relationship that could be construed as a potential conflict of interest.

## Footnotes

1 Note that one difference in the current data is a considerably longer control RT value (214 ms) as compared to Marinovic et al. (2013; Fig.1A, ∼135 ms), which may have been due to the size or luminosity of the visual IS. As our visual RT control value is within the expected range and given the similarity of experimental setup and instructions, this discrepancy is unlikely to reflect a different in preparatory state of the participants.

## References

[b1] Alibiglou L, MacKinnon CD (2012). The early release of planned movement by acoustic startle can be delayed by transcranial magnetic stimulation over motor cortex. J. Physiol.

[b2] Blumenthal TD, Cuthbert BN, Filion DL, Hackley S, Lipp OV, Van Boxtel A (2005). Committee report: guidelines for human startle eyeblink electromyographic studies. Psychophysiology.

[b3] Brown P, Rothwell JC, Thompson PD, Britton TC, Day BL, Marsden CD (1991). New observations on the normal auditory startle reflex in man. Brain.

[b4] Carlsen AN, Mackinnon CD (2010). Motor preparation is modulated by the resolution of the response timing information. Brain Res.

[b5] Carlsen AN, Chua R, Inglis JT, Sanderson DJ, Franks IM (2004a). Can prepared responses be stored subcortically?. Exp. Brain Res.

[b6] Carlsen AN, Chua R, Inglis JT, Sanderson DJ, Franks IM (2004b). Prepared movements are elicited early by startle. J. Mot. Behav.

[b7] Carlsen AN, Dakin CJ, Chua R, Franks IM (2007). Startle produces early response latencies that are distinct from stimulus intensity effects. Exp. Brain Res.

[b8] Carlsen AN, Chua R, Inglis JT, Sanderson DJ, Franks IM (2009). Differential effects of startle on reaction time for finger and arm movements. J. Neurophysiol.

[b9] Carlsen AN, Lam MY, Maslovat D, Chua R (2011a). Reaction time effects due to imperative stimulus modality are absent when a startle elicits a pre-programmed action. Neurosci. Lett.

[b10] Carlsen AN, Maslovat D, Lam MY, Chua R, Franks IM (2011b). Considerations for the use of a startling acoustic stimulus in studies of motor preparation in humans. Neurosci. Biobehav. Rev.

[b11] Carlsen AN, Maslovat D, Franks IM (2012). Preparation for voluntary movement in healthy and clincial populations: evidence from startle. Clin. Neurophysiol.

[b12] Davis M, Eaton RC (1984). The mammalian startle response. Neural mechanisms of startle behavior.

[b13] Gielen SC, Schmidt RA, Van den Heuvel PJ (1983). On the nature of intersensory facilitation of reaction time. Percept. Psychophys.

[b14] Grice GR (1968). Stimulus intensity and response evocation. Psychol. Rev.

[b15] Hanes DP, Schall JD (1996). Neural control of voluntary movement initiation. Science.

[b16] Honeycutt CF, Perreault EJ (2012). Planning of ballistic movement following stroke: insights from the startle reflex. PLoS ONE.

[b17] Honeycutt CF, Kharouta M, Perreault EJ (2013). Evidence for reticulospinal contributions to coordinated finger movements in humans. J. Neurophysiol.

[b18] Kohfeld DL (1971). Simple reaction time as a function of stimulus intensity in decibels of light and sound. J. Exp. Psychol.

[b19] Kumru H, Valls-Solé J (2006). Excitability of the pathways mediating the startle reaction before execution of a voluntary movement. Exp. Brain Res.

[b20] Li X, Aruin A (2005). Muscle activity onset time detection using teager-kaiser energy operator. Conf. Proc. IEEE Eng. Med. Biol. Soc.

[b21] MacKinnon CD, Bissig D, Chiusano J, Miller E, Rudnick L, Jager C (2007). Preparation of anticipatory postural adjustments prior to stepping. J. Neurophysiol.

[b22] Marinovic W, de Rugy A, Lipp OV, Tresilian JR (2013). Responses to loud auditory stimuli indicate that movement-related activation builds up in anticipation of action. J. Neurophysiol.

[b23] Marinovic W, de Rugy A, Riek S, Tresilian JR (2014). The early release of actions by loud sounds in muscles with distinct connectivity. Exp. Brain Res.

[b24] Maslovat D, Hodges NJ, Chua R, Franks IM (2011). Motor preparation and the effects of practice: evidence from startle. Behav. Neurosci.

[b25] Maslovat D, Chua R, Spencer HC, Forgaard CJ, Carlsen AN, Franks IM (2013). Evidence for a response preparation bottleneck during dual-task performance: effect of a startling acoustic stimulus on the psychological refractory period. Acta Psychol. (Amst).

[b26] Maslovat D, Carter MJ, Kennefick M, Carlsen AN (2014a). Startle neural activity is additive with normal cortical initiation-related activation. Neurosci. Lett.

[b27] Maslovat D, Klapp ST, Jagacinski RJ, Franks IM (2014b). Control of response timing occurs during the simple reaction time interval but on-line for choice reaction time. J. Exp. Psychol. Hum. Percept. Perform.

[b28] Miller J (1982). Divided attention: evidence for coactivation with redundant signals. Cogn. Psychol.

[b29] Nickerson RS (1973). Intersensory facilitation of reaction time: energy summation or preparation enhancement?. Psychol. Rev.

[b30] Ravichandran VJ, Honeycutt CF, Shemmell J, Perreault EJ (2013). Instruction-dependent modulation of the long-latency stretch reflex is associated with indicators of startle. Exp. Brain Res.

[b31] Thickbroom GW, Byrnes ML, Sacco P, Ghosh S, Morris IT, Mastaglia FL (2000). The role of the supplementary motor area in externally timed movement: the influence of predictability of movement timing. Brain Res.

[b32] Tresch UA, Perreault EJ, Honeycutt CF (2014). Startle evoked movement is delayed in older adults: implications for brainstem processing in the elderly. Physiol. Rep.

[b33] Valls-Sole J, Valldeoriola F, Tolosa E, Nobbe F (1997). Habituation of the auditory startle reaction is reduced during preparation for execution of a motor task in normal human subjects. Brain Res.

[b34] Valls-Solé J, Sole A, Valldeoriola F, Munoz E, Gonzalez LE, Tolosa ES (1995). Reaction time and acoustic startle in normal human subjects. Neurosci. Lett.

[b35] Valls-Solé J, Rothwell JC, Goulart FR, Cossu G (1999). Patterned ballistic movements triggered by a startle in healthy humans. J. Physiol.

[b36] Valls-Solé J, Kumru H, Kofler M (2008). Interaction between startle and voluntary reactions in humans. Exp. Brain Res.

[b37] Woodworth RS (1938). Experimental psychology.

